# Elevation of Peripheral *BDNF* Promoter Methylation Links to the Risk of Alzheimer's Disease

**DOI:** 10.1371/journal.pone.0110773

**Published:** 2014-11-03

**Authors:** Lan Chang, Yunliang Wang, Huihui Ji, Dongjun Dai, Xuting Xu, Danjie Jiang, Qingxiao Hong, Huadan Ye, Xiaonan Zhang, Xiaohui Zhou, Yu Liu, Jinfeng Li, Zhongming Chen, Ying Li, Dongsheng Zhou, Renjie Zhuo, Yuzheng Zhang, Honglei Yin, Congcong Mao, Shiwei Duan, Qinwen Wang

**Affiliations:** 1 Zhejiang Provincial Key Laboratory of Pathophysiology, School of Medicine, Ningbo University, Ningbo, Zhejiang, China; 2 Department of Neurology, the 148 Central Hospital of PLA, Zibo, Shandong, China; 3 Department of Internal Medicine for Cadres, the First Affiliated Hospital of Xinjiang Medical University, Urumchi, China; 4 Ningbo Kangning Hospital, Zhejiang, China; 5 Ningbo No. 1 Hospital, Zhejiang, China; Università di Napoli Federico II, Italy

## Abstract

Brain derived neurotrophic factor (BDNF) has been known to play an important role in various mental disorders or diseases such as Alzheimer's disease (AD). The aim of our study was to assess whether *BDNF* promoter methylation in peripheral blood was able to predict the risk of AD. A total of 44 AD patients and 62 age- and gender-matched controls were recruited in the current case-control study. Using the bisulphite pyrosequencing technology, we evaluated four CpG sites in the promoter of the *BDNF*. Our results showed that *BDNF* methylation was significantly higher in AD cases than in the controls (CpG1: *p* = 10.021; CpG2: *p* = 0.002; CpG3: *p* = 0.007; CpG4: *p* = 0.005; average methylation: *p* = 0.004). In addition, *BDNF* promoter methylation was shown to be significantly correlated with the levels of alkaline phosphatase (ALP), glucose, Lp(a), ApoE and ApoA in males (ALP: r = −0.308, *p* = 0.042; glucose: r = −0.383, *p* = 0.010; Lp(a): r = 0.333, *p* = 0.027; ApoE: r = −0.345, *p* = 0.032;), ApoA levels in females (r = 0.362, *p* = 0.033), and C Reactive Protein (CRP) levels in both genders (males: r = −0.373, *p* = 0.016; females: r = −0.399, *p* = 0.021). Our work suggested that peripheral *BDNF* promoter methylation might be a diagnostic marker of AD risk, although its underlying function remains to be elaborated in the future.

## Introduction

Alzheimer's disease (AD) is a neurodegenerative disorder characterized by progressive memory disorder and cognitive dysfunction [1]. The prevalence of AD was 26.6 million in 2006, and the number is expected to quadruple in 2050, causing a huge burden on both family and society [2]. AD is a complex disease affected by both environmental and genetic factors [3]. Twin studies showed that approximately 80% of cases resulted from inheritance [4]. Although a handful of genetic markers have been identified [5], the pathogenesis of AD remains unclear. Environmental factors were also shown to be related to AD [6].

As a link between genetic and environmental factors, epigenetic modification is able to cause stunted growth, mental retardation, feminization, and other complex syndromes [7], [8]. Genes with aberrant DNA methylation could change gene expression levels [9], and thus, might contribute to the risk of diseases or disorders such as coronary heart disease [10], [11], essential hypertension [12], schizophrenia [9], leukemia [13] and type 2 diabetes [7], [14]. A global hypermethylation was found in the AD middle frontal gyrus and middle temporal gyrus with no apparent influence of gender, age, postmortem delay, or tissue storage time [15]. A decreased global DNA methylation level was also found in the hippocampus of AD patients [16].

The brain derived neurotrophic factor (BDNF) gene is located on chromosome 11p13, encoding a secretory protein of the neurotrophic factor family [17]. *BDNF* was shown to protect neurons from various attacks [18], and it was associated with several psychiatric disorders such as substance-related disorders, eating disorders, and schizophrenia [19]. Reduction in *BDNF*-immunoreactive cell bodies was found in AD patients [20]. A significantly higher *BDNF* promoter methylation level was found in the male schizophrenic patients [21] and in the depressive patients with suicidal behavior [22]. Prenatal stress was shown to induce decreased *BDNF* expression and increased methylation of the *BDNF* gene body in rats [23]. An increased level of *BDNF* promoter methylation and a decreased level of *BDNF* mRNA were simultaneously observed in the AD brain [24]. *BDNF* was also treated as a new target in the AD treatment [25]. In this study, we measured *BDNF* promoter methylation levels in peripheral blood to explore its association with AD in the Han Chinese population.

## Methods and Materials

A total of 44 sporadic AD cases and 62 matched controls were selected from Ningbo No. 1 Hospital and Ningbo Kangning Hospital. AD cases were diagnosed by experienced neurological physicians (CZ and ZQ) according to ICD-10 criteria, and confirmed by the evidence that comprised their medical and family histories, neurological examination, outcomes of blood tests, brain imaging examination (computed tomography or magnetic resonance), neuropsychological tests, as well as cognitive screening tests, including mini-mental state. No familial AD cases were included in the current study. At the time of sample collection, all the controls had been assessed to be free from any kind of disorder. All the individuals were Han Chinese originating from Ningbo city in Eastern China, and their characteristics are as described in Table 1. Blood samples were collected in 3.2% citrate sodium-treated tubes and then stored at −80°C. The study protocol was approved by the Ethical Committees of Ningbo University, Ningbo No. 1 Hospital and Ningbo Kangning Hospital. Written informed consents were obtained from all the subjects through themselves or their guardians.

**Table 1:** Characteristics of 106 subjects.

Characteristics	All subjects (n = 106) Mean ± SD.	Range (Overall)
		
Age	79.78±8.27	[53–96]
Onset age	74.42±11.35	[50–96]
Course of disease	7.02±5.30	[0–20]
Hypertension (Yes/No)	71/35	/
Smoking (Yes/No)	17/89	/
Diabetes (Yes/No)	32/74	/
Drugs (Memantine/Exelon/Aricept)	17/3/15	/
CpG1 (%)	10.69±4.26	[5]–[38]
CpG2 (%)	5.22±3.25	[1]–[29]
CpG3 (%)	8.43±3.91	[2]–[25]
CpG4 (%)	8.87±4.06	[0–26]
Mean *BDNF* methylation (%)	8.30±3.68	[2.5–29.5]

The content of serum total protein (TP) was measured by the biuret method [26], and serum albumin (ALB) was tested by the bromocresol green method [27]. Plasma levels of glutamic-pyruvic transaminase (ALT), alkaline phosphatase (ALP) and glutamic oxalacetic transaminase (AST) were determined by the velocity method [28], [29]. The levels of total bile acid (TBA) and homocysteine (Hcy) were measured by the cycling enzymatic method [30], [31]. The concentrations of blood glucose (Glu), triglyceride (TG), total cholesterol (TC), carbamide (UREA), creatinine (CRE) and uric acid (UA) in plasma were determined using the classic enzymatic methods [32]–[37]. The high-density lipoprotein cholesterol (HDL-C) level was determined by the one-step detection method [38]. The proportion of apolipoprotein A (ApoA) and apolipoprotein B (ApoB) were measured by turbidimetry [39], [40]. The content of lipoprotein A (Lp(a)) was detected using the endpoint method [41]. C Reactive Protein (CRP) and apolipoprotein E (ApoE) were measured by using a latex agglutination assay [42] and an immunoturbidimetric assay [43], respectively.

Human genomic DNA was extracted from peripheral blood samples using the nucleic acid extraction analyzer (Lab-Aid 820, Xiamen City, China). DNA concentrations were determined by using the ultramicro nucleic acid ultraviolet tester (NanoDrop 2000, Wilmington, USA). DNA methylation was measured by using pyrosequencing technology, which combines sodium bisulfite DNA conversion chemistry (EZ DNA Methylation-GoldTM Kit; ZYMO RESEARCH), polymerase chain reaction (PCR) amplification (Zymo TaqTM PreMix, ZYMO RESEARCH) and sequencing by synthesis assay (Pyromark Gold Q24 Reagents; Qiagen) of the CGI region on *BDNF* promoter. PCR primers were designed by PyroMark Assay Design software. Sequences of the PCR primers were shown in Table S1.

All of the statistical analyses were performed by Statistical Program for Social Sciences (SPSS) software 16.0 (SPSS, Inc., Chicago, IL, USA) and a *p* value <0.05 was considered to be significant. Two independent samples t-test was used to compared differences in the mean values of continuous variables between the AD cases and controls. The associations between *BDNF* methylation and metabolic characteristics of AD subjects were assessed by Pearson's correlation test. Bonferroni correction was used to adjust our results.

## Results

**Figure 1:**
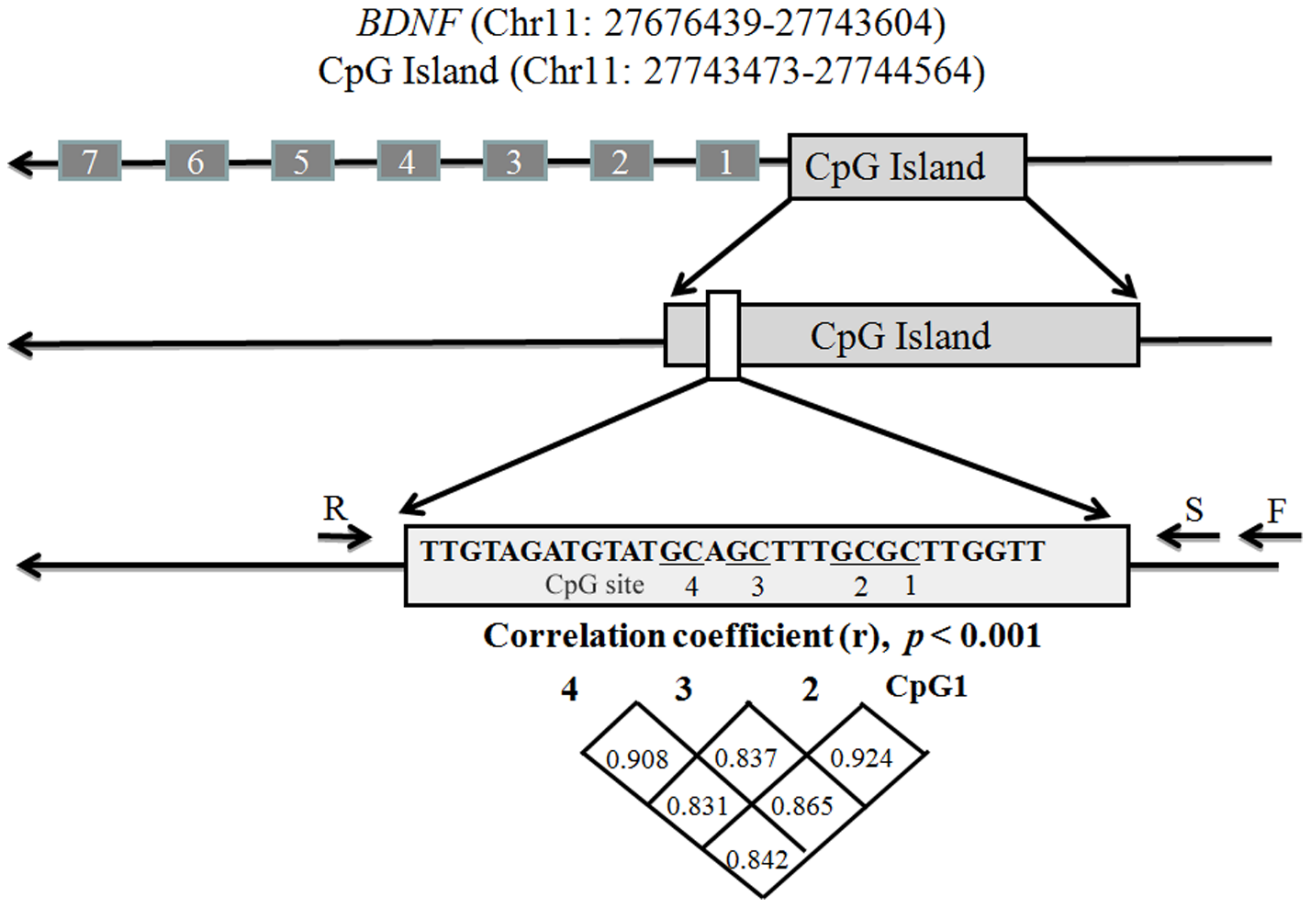
Correlation among 4 CpGs in *BDNF* promoter. F: Forward primer, R: Reverse primer, S: Sequencing primer.

As shown in Figure 1, a total of four CpGs on a fragment in the *BDNF* promoter were included in this specific methylation assay. Methylation levels of the four CpGs were significantly correlated with each other (Figure 1, r>0.8, *p*<0.001). Therefore, association tests were performed for each of the four CpGs as well as their average methylation.

**Figure 2:**
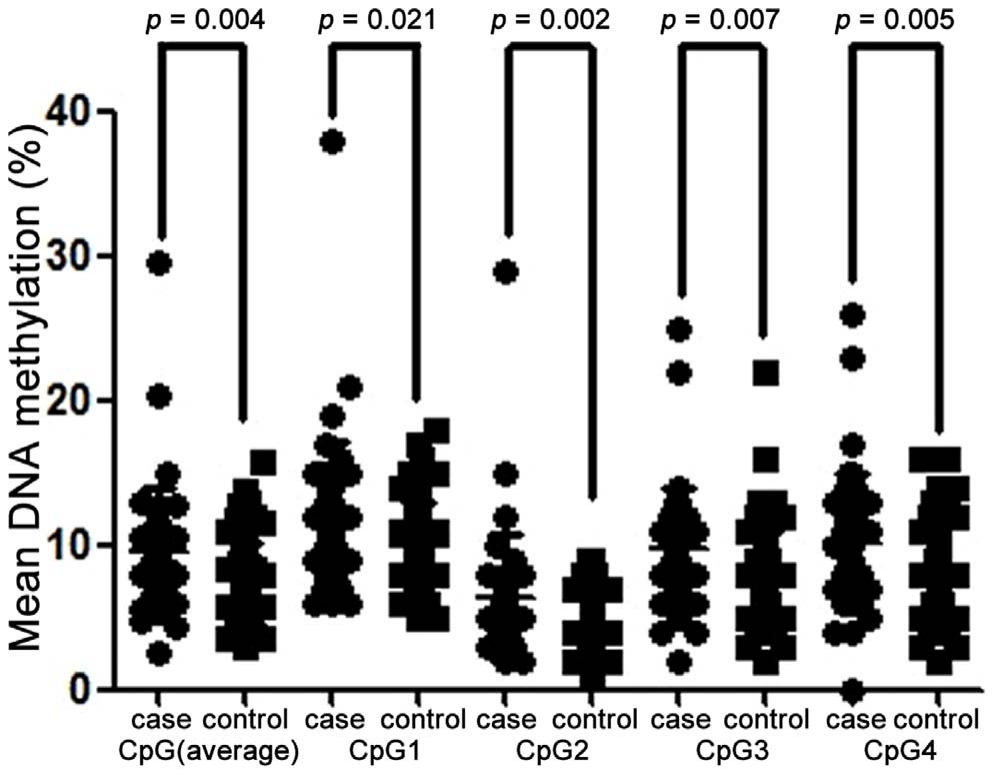
Comparison of *BDNF* methylation levels between cases and controls.

As shown in Table 2 and Figure 2, *BDNF* promoter methylation was significantly elevated in the AD cases as opposed to the controls (CpG1: *p* = 0.021; CpG2: *p* = 0.002; CpG3: *p* = 0.007; CpG4: *p* = 0.005; average methylation: *p* = 0.004). A further subgroup analysis by gender showed significant associations with AD for CpG2 methylation in males (*p* = 0.018, Table 2) and CpG4 methylation in females (*p* = 0.039, Table 2). The lack of association for the other tests might be due to a lack of power in the subgroup analyses by gender. In addition, our results showed that there was no significant interaction between AD drugs and *BDNF* methylation or individual CpG sites (*p*>0.05, Figure S1 and Table S2).

**Table 2:** Comparisons of *BDNF* methylation levels between cases and controls.

Characteristics	Case Mean ± SD	Control Mean ± SD	*p* value
All			
CpG1 (%)	11.82±5.32	9.89±3.13	**0.021**
CpG2 (%)	6.39±4.27	4.39±1.92	**0.002#**
CpG3 (%)	9.64±4.24	7.58±3.45	**0.007**
CpG4 (%)	10.16±4.69	7.95±3.29	**0.005**
Mean *BDNF* methylation (%)	9.50±4.43	7.45±2.77	**0.004#**
Male			
CpG1 (%)	10.85±2.87	9.87±2.72	0.191
CpG2 (%)	5.60±1.98	4.42±1.73	**0.018**
CpG3 (%)	8.25±2.53	7.40±2.79	0.249
CpG4 (%)	8.80±2.91	7.96±3.07	0.303
Mean *BDNF* methylation (%)	8.38±2.46	7.41±2.39	0.142
Female			
CpG1 (%)	12.62±6.67	9.94±4.12	0.150
CpG2 (%)	7.04±5.47	4.29±2.42	0.060
CpG3 (%)	10.79±5.02	8.06±4.85	0.090
CpG4 (%)	11.29±5.58	7.94±3.90	**0.039**
Mean *BDNF* methylation (%)	10.44±5.45	7.56±3.67	0.066

* *p* value less than or equal to 0.05 is in bold.

#: *p* value survived after multiple testing.

**Figure 3:**
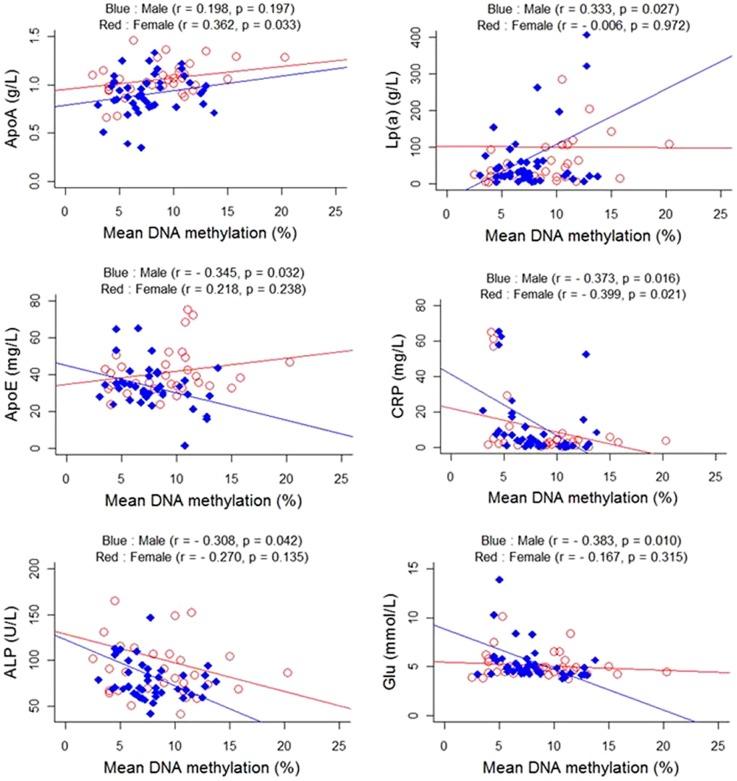
Correlation analyses between the *BDNF* promoter methylation levels and the metabolic features of samples.

A total of 22 phenotypes were collected for all of the involved samples (Table 3). Significantly higher levels of ALB (*p* = 0.045), ApoA (*p* = 0.006), Lp(a) (*p* = 1.68E-04) and Hcy (*p* = 0.045) were found in the AD cases. A significantly lower CRP level (*p* = 0.014) was also found in the AD cases. As shown in Figure 3, further subgroup tests by gender showed that male-specific associations were only found between *BDNF* promoter methylation and ALP (r = −0.308; *p* = 0.042), glucose (r = −0.383; *p* = 0.010), Lp(a) (r = 0.333; *p* = 0.027), and ApoE (r = −0.345; *p* = 0.032). Significant association between *BDNF* promoter methylation and ApoA was only found in females (r = 0.362; *p* = 0.033). Otherwise, we observed CRP was significantly associated with *BDNF* promoter methylation in both genders (males: r = −0.373, *p* = 0.016; females: r = −0.399, *p* = 0.021).

**Table 3:** Characteristics of subjects from cases and controls.

Characteristics	Case (n = 44)	Control (n = 62)	*p* value
	Mean ± SD	Mean ± SD	
Age (years)	80.00±8.92	79.63±7.85	0.821
TP (g/L)	68.74±6.80	65.77±9.58	0.135
ALB (g/L)	38.43±3.82	36.65±3.89	**0.045**
GLB (g/L)	30.31±5.30	30.03±5.70	0.830
A/G	1.31±0.22	1.27±0.23	0.432
ALT (U/L)	13.81±10.51	18.12±13.34	0.183^a^
ALP (U/L)	78.96±24.27	96.33±62.80	0.147^a^
TBA (µmol/L)	6.80±3.81	5.96±5.85	0.499
AST (U/L)	20.75±7.19	23.41±11.64	0.348^a^
Glu (mmol/L)	5.18±1.57	5.51±2.69	0.359^b^
TG (mmol/L)	1.34±0.77	1.41±0.97	0.896^a^
TC (mmol/L)	4.43±1.04	4.25±1.24	0.430
HDL-C (mmol/L)	1.06±0.20	1.03±0.30	0.136
ApoA (g/L)	1.06±0.21	0.93±0.20	**0.006**
ApoB (g/L)	0.66±0.18	0.72±0.26	0.235
Lp(a) (g/L)	179.19±231.22	34.56±27.13	**1.68E-04^a^**
ApoE (mg/L)	37.73±17.44	36.69±10.37	0.800
UREA (mmol/L)	7.72±9.84	6.45±3.42	0.778^b^
CRE (µmol/L)	82.09±46.59	78.83±29.82	0.700
UA (µmol/L)	308.39±104.87	308.41±111.66	0.999
Hcy (µmol/L)	19.76±10.82	17.64±20.63	**0.045^a^**
CRP (mg/L)	6.20±11.72	14.84±25.97	**0.014^a^**
Mean *BDNF* methylation (%)	9.50±4.43	7.45±2.77	**0.004**

* *p* value less than or equal to 0.05 is in bold. a: Log-transformation was used. b: Nonparametric rank test was applied. TP: total protein; ALB: serum albumin; GLB: serum globulin; A/G: ALB/GLB; ALT: glutamic-pyruvic transaminase; ALP: alkaline phosphatase; TBA: total bile acid; AST: glutamic oxalacetic transaminase; Glu: blood glucose; TG: triglyceride; TC: total cholesterol; HDL-C: high-density lipoprotein cholesterol; ApoA: apolipoprotein A; ApoB: apolipoprotein B; Lp(a): lipoprotein A; ApoE: apolipoprotein E; UREA: carbamide; CRE: creatinine; UA: uric acid; Hcy: homocysteine; CRP: C Reactive Protein.

Age is an important factor for AD, however, our correlation tests showed a lack of association between *BDNF* methylation and age (Table S3). A breakdown analysis by gender also found no association of age with all CpGs in both gender subgroups. Also, no association was found between *BDNF* methylation and the onset of increased age in the all samples and subgrouped samples by gender (Table S4).

## Discussion

About 40% of promoters in mammalian genes were hypomethylated [44] and promoter hypermethylation often silences gene expression [45]. Aberrant promoter methylation exists in several diseases such as essential hypertension [12], type 2 diabetes [14], schizophrenia [9], coronary heart disease [9], [10], leukemia [13], and colorectal cancer [46]. Higher *DUSP22* promoter methylation was found in AD brain tissues [47]. Lower *SORL1* promoter methylation was found in the brain and blood of AD patients [48]. Our study showed there was a significantly elevated methylation of the *BDNF* promoter in peripheral blood. Our findings agreed to the previous observation that higher *BDNF* promoter methylation was found in the brain tissues of AD patients [24] and gave a new hint for the diagnosis of AD using the peripheral blood as a surrogate.

Decreased *BDNF* promoter methylation levels were shown to be correlated with increased *BDNF* mRNA and protein expression in the epileptic hippocampus [49]. Reductions of BDNF protein in frozen postmortem AD frontal cortex samples compared to controls showed reduced mRNA levels of *BDNF*, which might be related to the hypermethylated *BDNF* promoter in the same tissues [24]. Decreased *BDNF* expression in the amygdala and hippocampus of prenatally stressed rats, both at weaning and in adulthood, was found to be accompanied by increased *BDNF* gene body methylation [23]. The elevation of *BDNF* promoter methylation in AD peripheral blood might indicate a decreased *BDNF* expression, although future work is needed to confirm our speculation.

Gender disparities are widely shown in AD. Women are shown to be at a higher risk for AD in all the age stratums and the age-adjusted odds ratio for women was 3.1 between AD cases and controls [50]. Meta-analyses of 16 human case-control studies observed significant associations between *BNDF* Val66Met and AD in females, but not in males [51]. Animal testing with aged mice found a higher level of the *BDNF* gene in female mice, but not in male mice, and female mice were more sensitive to kainic acid-induced excitotoxicity, which can lead to hippocampal neurodegeneration [52]. Previous studies also indicated that gender-specific DNA methylation existed in mice [53] and humans [10], [54]. In this study, we observed that methylation of all four CpGs were significantly elevated in AD patients than in controls. In the subgroup analyses by gender, our results showed that *BDNF* CpG2 and CpG4 methylation was significantly higher in male and female AD cases, respectively, and that a trend towards a significant result was found in females for average methylation and CpG2. Our work suggests that *BDNF* promoter methylation might have gender dimorphism in the association of *BDNF* methylation with AD. Further studies with larger samples need to be done to confirm our observation.

A total of 22 phenotypes were analyzed among our subjects. A significant association of AD was found for ApoA, which plays a role in cholesterol transport and the regulation of inflammation [55] as well as affects Aβ aggregation and deposition [56]. A previous study showed that serum ApoA concentration was highly correlated with the severity of AD [57]. A significant association of AD was found with Lp(a), which was shown to be related to dementia [58] and AD [59]. A significant association of AD with ALB was found in our study. Human serum ALB could indicate AD by regulating Aβ peptide fiber growth in the brain interstitium [60]. A significantlyhigher level of Hcy was also found in this present study. A higher level of Hcy was a risk factor of AD [61] and may cause learning and memory deficits by generating reactive oxygen species [62]. CRP involved in the systemic response to inflammation [63], and our results were consistent with an earlier study which found reduced levels of plasma CRP in AD [64].

Gender-stratified correlation analyses were also performed between *BDNF* promoter methylation and the 22 phenotypes. Among them, we observed four significant associations in males, a significant association of ApoA in females, and a significant association of CRP in both genders. Our results suggest that *BDNF* promoter methylation might influence the pathophysiology of AD through its influence on those factors. This might also provide new hints to elucidate the molecular mechanisms in AD pathogenesis.

There are several limitations of our study that need to be taken into consideration. Firstly, there were only 44 cases and 62 controls in our study. The moderate number of samples may have influenced the results of our study, especially for the gender-stratified association test of *BDNF* methylation with AD. Secondly, although the best-scored primers harbor a fragment with 4 CpGs which might not fully represent the overall contribution of *BDNF* methylation to AD patients. Further studies of other CpGs in the promoter and gene body are needed. Thirdly, since we could not obtain the brain tissues, we only tested the DNA methylation levels of *BDNF* in peripheral blood. Further comprehensive studies are needed to test the concordance of *BDNF* methylation between brain tissues and peripheral blood. Fourthly, aged persons have a tendency to get different illnesses. Although we tried our best to avoid potential factors when we matched the cases and controls, unknown factors exist in the samples that might influence the results of our work. Fifthly, we assessed four CpG positions per pyrosequencing read, so some *p* values might not retain their significance after being corrected by the number of CpG sites. A chance of random positive findings could not be excluded. We marked the *p* values that remained significant after multiple test corrections. We kept the uncorrected *p* values in the tables and annotated some with the correction methods for the readers' reference.

## Conclusions

Our study suggested that there was a significant contribution of *BDNF* promoter methylation to the risk of AD. Aberrant *BDNF* methylation in peripheral blood could serve as a surrogate for the diagnosis of AD. In addition, our study also found that *BDNF* methylation was associated with several biomedical factors, which consisted of ALP, Glucose, Lp(a) and ApoE in males, ApoA in females and CRP in both genders.

## Supporting Information

Figure S1 Comparison of *BDNF* promoter methylation among AD patients with different drug treatment.
(TIF)

Table S1 Primers for *BDNF* methylation analysis.
(DOC)

Table S2 Correlation analyses between *BDNF* promoter methylation levels and AD drugs.
(DOC)

Table S3 Correlation analyses between *BDNF* promoter methylation levels and age in total, males and females samples.
(DOC)

Table S4 Correlation analyses between *BDNF* promoter methylation levels and onset age in total, males and females samples.
(DOC)
