# Supplementary figures and images for: Elevation of Peripheral BDNF Promoter Methylation Links to the Risk of Alzheimer's Disease
Source: PLoS One. 2014 Nov 3;9(11):e110773. doi: 10.1371/journal.pone.0110773 (PMC4217733; doi:10.1371/journal.pone.0110773)

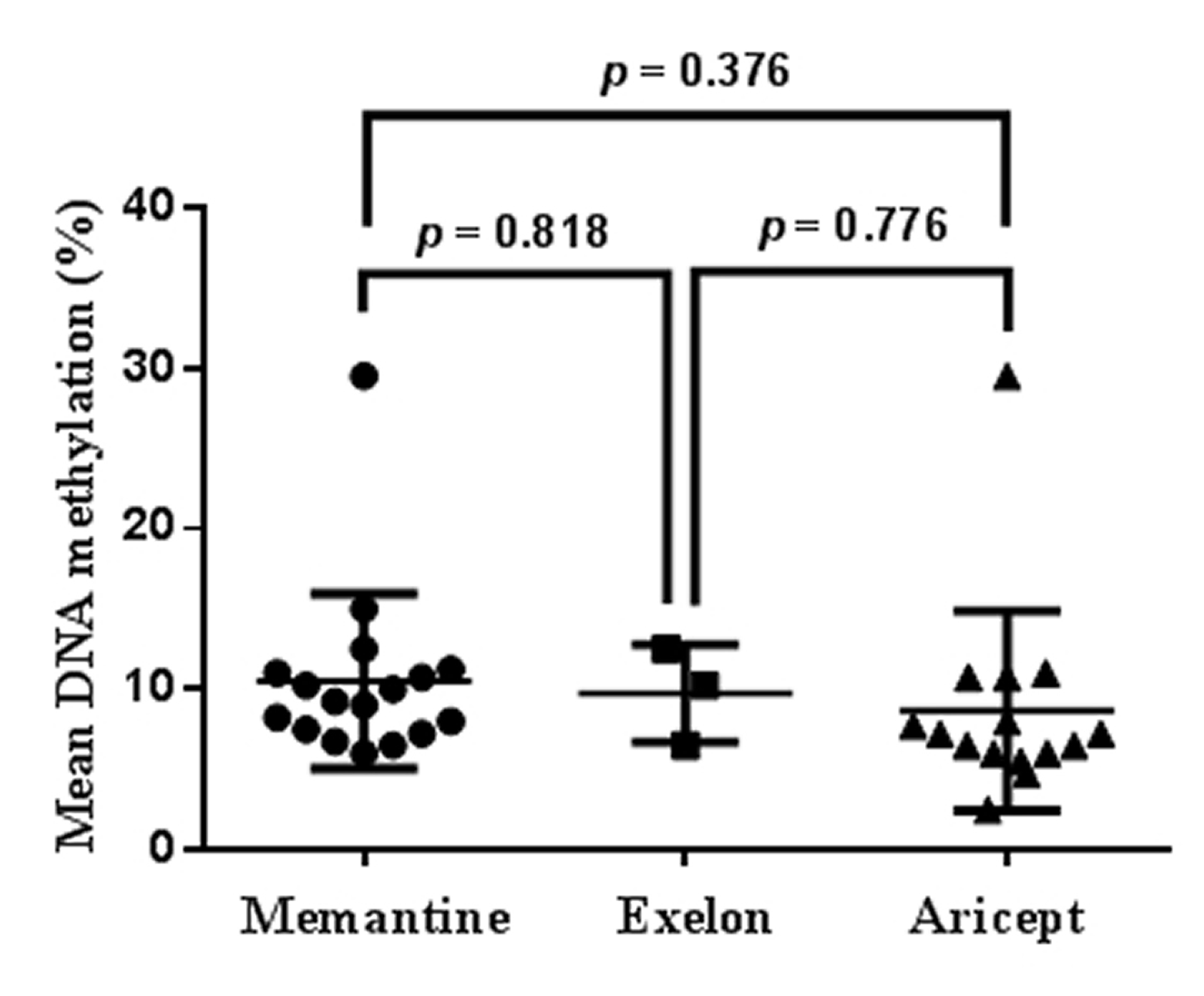

Supplement: Figure S1 — Comparison of BDNF promoter methylation among AD patients with different drug treatment. (TIF) [file pone.0110773.s001.tif]
