# Supplementary material for: Elevation of Peripheral BDNF Promoter Methylation Links to the Risk of Alzheimer's Disease
Source: PLoS One. 2014 Nov 3;9(11):e110773. doi: 10.1371/journal.pone.0110773 (PMC4217733; doi:10.1371/journal.pone.0110773)
Supplement: Table S1 — Primers for BDNF methylation analysis. (DOC) [file pone.0110773.s002.doc]

Supplemental table 1: Primers for *BDNF* methylation analysis

| Forward primer: | 5’-TTAGTATTTAAGAGGAAAAGGGAAAGTTGT-3’ |
| --- | --- |
| Reverse primer: | 5’-Biotin-CCCCCATCATAACTAAAAATCT-3’ |
| Sequencing primer: | 5’-GGGAAAGTTGTTGGG-3’ |
