# Supplementary material for: Elevation of Peripheral BDNF Promoter Methylation Links to the Risk of Alzheimer's Disease
Source: PLoS One. 2014 Nov 3;9(11):e110773. doi: 10.1371/journal.pone.0110773 (PMC4217733; doi:10.1371/journal.pone.0110773)
Supplement: Table S2 — Correlation analyses between BDNF promoter methylation levels and AD drugs. (DOC) [file pone.0110773.s003.doc]

Supplemental table 2: Correlation analyses between *BDNF* promoter methylation levels and AD drugs.

|  | Memantine & Exelon | Memantine & Aricept | Exelon & Aricept |
| --- | --- | --- | --- |
| Average CpG1-4 | *p* = 0.818 | *p* = 0.376 | *p* = 0.776 |
| CpG1 | *p* = 0.784 | *p* = 0.451 | *p* = 0.859 |
| CpG2 | *p* = 0.897 | *p* = 0.570 | *p* = 0.840 |
| CpG3 | *p* = 0.752 | *p* = 0.235 | *p* = 0.713 |
| CpG4 | *p* = 0.851 | *p* = 0.275 | *p* = 0.673 |
