# Supplementary material for: Elevation of Peripheral BDNF Promoter Methylation Links to the Risk of Alzheimer's Disease
Source: PLoS One. 2014 Nov 3;9(11):e110773. doi: 10.1371/journal.pone.0110773 (PMC4217733; doi:10.1371/journal.pone.0110773)
Supplement: Table S3 — Correlation analyses between BDNF promoter methylation levels and age in total, males and females samples. (DOC) [file pone.0110773.s004.doc]

Supplemental table 3: Correlation analyses between *BDNF* promoter methylation levels and age in total, males and females samples.

| Characteristics | r | *p* value |
| --- | --- | --- |
| All |  |  |
| CpG1 | 0.097 | 0.324 |
| CpG2 | 0.089 | 0.362 |
| CpG3 | 0.064 | 0.515 |
| CpG4 | 0.100 | 0.309 |
| Mean *BDNF* methylation | 0.092 | 0.346 |
| Male |  |  |
| CpG1 | 0.048 | 0.706 |
| CpG2 | 0.023 | 0.858 |
| CpG3 | 0.145 | 0.250 |
| CpG4 | 0.153 | 0.224 |
| Mean *BDNF* methylation | 0.106 | 0.400 |
| Female |  |  |
| CpG1 | 0.225 | 0.157 |
| CpG2 | 0.229 | 0.151 |
| CpG3 | 0.168 | 0.295 |
| CpG4 | 0.197 | 0.216 |
| Mean *BDNF* methylation | 0.214 | 0.178 |
