# Supplementary material for: Elevation of Peripheral BDNF Promoter Methylation Links to the Risk of Alzheimer's Disease
Source: PLoS One. 2014 Nov 3;9(11):e110773. doi: 10.1371/journal.pone.0110773 (PMC4217733; doi:10.1371/journal.pone.0110773)
Supplement: Table S4 — Correlation analyses between BDNF promoter methylation levels and onset age in total, males and females samples. (DOC) [file pone.0110773.s005.doc]

Supplemental table 4: Correlation analyses between *BDNF* promoter methylation levels and onset age in total, males and females samples.

| Characteristics | r | *p* value |
| --- | --- | --- |
| All |  |  |
| CpG1 | -0.209 | 0.179 |
| CpG2 | -0.16 | 0.304 |
| CpG3 | -0.368 | 0.015 |
| CpG4 | -0.343 | 0.024 |
| Mean *BDNF* methylation | -0.28 | 0.069 |
| Male |  |  |
| CpG1 | -0.489 | 0.033 |
| CpG2 | -0.368 | 0.121 |
| CpG3 | -0.253 | 0.295 |
| CpG4 | -0.38 | 0.109 |
| Mean *BDNF* methylation | -0.396 | 0.093 |
| Female |  |  |
| CpG1 | -0.079 | 0.713 |
| CpG2 | -0.057 | 0.792 |
| CpG3 | -0.334 | 0.111 |
| CpG4 | -0.259 | 0.221 |
| Mean *BDNF* methylation | -0.182 | 0.395 |
